# Structural Insights of the DciA Helicase Loader in Its Relationship with DNA

**DOI:** 10.3390/ijms24021427

**Published:** 2023-01-11

**Authors:** Claire Cargemel, Sonia Baconnais, Magali Aumont-Nicaise, Magali Noiray, Lia Maurin, Jessica Andreani, Hélène Walbott, Eric Le Cam, Françoise Ochsenbein, Stéphanie Marsin, Sophie Quevillon-Cheruel

**Affiliations:** 1 Université Paris-Saclay, CEA, CNRS, Institute for Integrative Biology of the Cell (I2BC), 91198 Gif-sur-Yvette, France; 2 Genome Integrity and Cancer UMR 9019 CNRS, Université Paris Saclay, Gustave Roussy, 114 Rue Edouard Vaillant, 94805 Villejuif, France

**Keywords:** DciA, DNA interaction, structural study, replicative helicase loading

## Abstract

DciA is the ancestral bacterial replicative helicase loader, punctually replaced during evolution by the DnaC/I loaders of phage origin. DnaC helps the helicase to load onto DNA by cracking open the hexameric ring, but the mechanism of loading by DciA remains unknown. We demonstrate by electron microscopy, nuclear magnetic resonance (NMR) spectroscopy, and biochemistry experiments that DciA, which folds into a KH-like domain, interacts with not only single-stranded but also double-stranded DNA, in an atypical mode. Some point mutations of the long α-helix 1 demonstrate its importance in the interaction of DciA for various DNA substrates mimicking single-stranded, double-stranded, and forked DNA. Some of these mutations also affect the loading of the helicase by DciA. We come to the hypothesis that DciA could be a DNA chaperone by intercalating itself between the two DNA strands to stabilize it. This work allows us to propose that the direct interaction of DciA with DNA could play a role in the loading mechanism of the helicase.

## 1. Introduction

The ability to replicate DNA is essential for the survival of organisms. The early initiation steps in bacteria are regulated by the opening of the *ori* site of the genomic DNA, thanks to the initiation protein DnaA, followed by the loading of two DnaB replicative helicases [1]. In bacteria encoding the *dnaC* gene, the closed hexameric ring of DnaB is cracked open following the attachment of six molecules of DnaC to the back of the helicase, forming a spiral structure in which the ssDNA can be accommodated [2,3,4]. Once the helicases are loaded onto the ssDNA, the replication machinery is recruited, inducing the synthesis of the new DNA strands [5].

However, it has been established that the presence of *dnaC* and its homolog *dnaI* (*dnaC/I*) in the bacterial world is an exception. These genes have been acquired late, seven times independently in the course of evolution, by the horizontal transfer of phage genes [6]. In the majority of bacterial phyla, the *dciA* gene is present instead of *dnaC/I*. The two genes are exclusive of each other, and they show no similarity either in sequence or in structure [7]. DnaC is composed of a C-terminal AAA+ ATPase domain and a long N-terminal α-helix whose end contacts the helicase [2]. DciA consists of a KH-like domain, the DUF721 domain, followed by a C-terminal unstructured domain, which adopts a small hairpin structure of two α-helices in contact with the helicase [7,8,9]. Recently, the conservation and the evolution of the DciA proteins in the entire living world have been studied by a computational evolutionary approach [10]. The DciA proteins vary in length and in the KH-like domain position and have been classified into four groups, based on the lengths of the flanking N- and C-terminal extensions forming unstructured linker motifs connecting α-helical structures to the KH-like domain. DciA from *Vibrio cholerae* (*Vc*DciA) belongs to group two, due to the presence of its unstructured C-terminal extension [7].

We previously studied the *Vc*DnaB•*Vc*DciA complex from a structural and biochemical point of view, and we showed that *Vc*DciA, like DnaC, targets the LH/DH module of the helicase. Indeed, the N-terminal extremity of DnaC and the hairpin-structured CTD of DciA form, respectively, a 3- or 5- helix bundle with the two-helix LH/DH module of DnaB [2,9,11]. Despite their structural difference, the two loaders thus probably perform the same function on the replicative helicase. This finding is reinforced by the fact that in vitro the two loaders can be exchanged for the loading of their non-cognate helicases [9]. However, the mechanism used by DciA to load the DnaB helicase is not yet fully understood. High-resolution crystallographic structure determination of the *Vc*DnaB•*Vc*DciA complex has proposed that DciA alone is probably not capable of opening the closed planar ring of DnaB on the DnaC model [9], leaving open the question of its mode of operation.

On the opposite face of DciA contacting the LH/DH module of DnaB, its NTD KH-fold domain points outward from the ring structure of the helicase and is exposed to the solvent [9]. It is therefore potentially available to interact with another partner, which could be a protein or a nucleic acid. The KH fold is described in the literature as often interacting with nucleic acids, especially with ssDNA or RNA [12,13,14]. We investigate, in this paper, the ability of DciA from *Vibrio cholerae* to interact with DNA and whether the loading mechanism by DciA could involve its interplay with DNA.

## 2. Results and Discussion

### 2.1. VcDciA Binds to Single- and Double-Stranded Oligodeoxynucleotide Substrates

**Figure 1:**
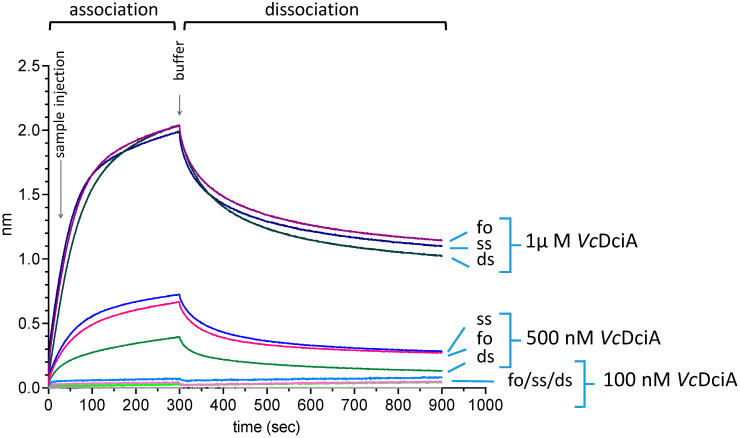
Interaction of *Vc*DciA with single- and double-stranded DNA, and with a fork-mimicking DNA, using BLI technology. An 80-base oligonucleotide (ssDNA) was attached via its biotinylated 5′ end to the chip before or after a prior hybridization step with complementary strands, to form the forked DNA (foDNA) or the double-stranded DNA (dsDNA) substrates (see Section 3). *Vc*DciA was injected at 3 concentrations on these 3 substrates in a buffer solution containing ATP.

DciA is a small protein, which has the particularity of a high isoelectric point, ranging from 10 to 12, depending on the bacterial origin. DciA from *Vibrio cholerae* (*Vc*DciA) shows a pI of 10.04, due to the presence of 13 arginines and 13 lysines over 158 residues. This led us to wonder whether *Vc*DciA, which harbors a KH-fold domain often found in the literature to interact with nucleic acids, can interact with DNA. For this purpose, we used the BLI technique to check whether *Vc*DciA was able to bind to single-stranded oligodeoxynucleotides (ssDNA) as well as double-stranded DNA (dsDNA) and a DNA substrate mimicking a replication fork (foDNA; Section 3 and Table 1). The integrity of the substrates was verified by testing their interaction with SSB, which is a protein that binds only to ssDNA (Supplementary Figure S1) [15]. Whereas SSB discriminated among the three different substrates as expected, *Vc*DciA showed similar affinity for all of them (Figure 1). The dissociation curve profiles showed a fast phase followed by a slower phase, suggesting that there are two types of interactions between *Vc*DciA and DNA, and the fitting of the curves using Langmuir Isotherm was incorrect. (Supplementary Figure S2). The interaction properties of *Vc*DciA for DNA do not seem to fall within classical 1:1 models of DNA–protein interaction.

### 2.2. VcDciA Binds to Single- and Double-Stranded DNA in Intermediate Structures

**Figure 2:**
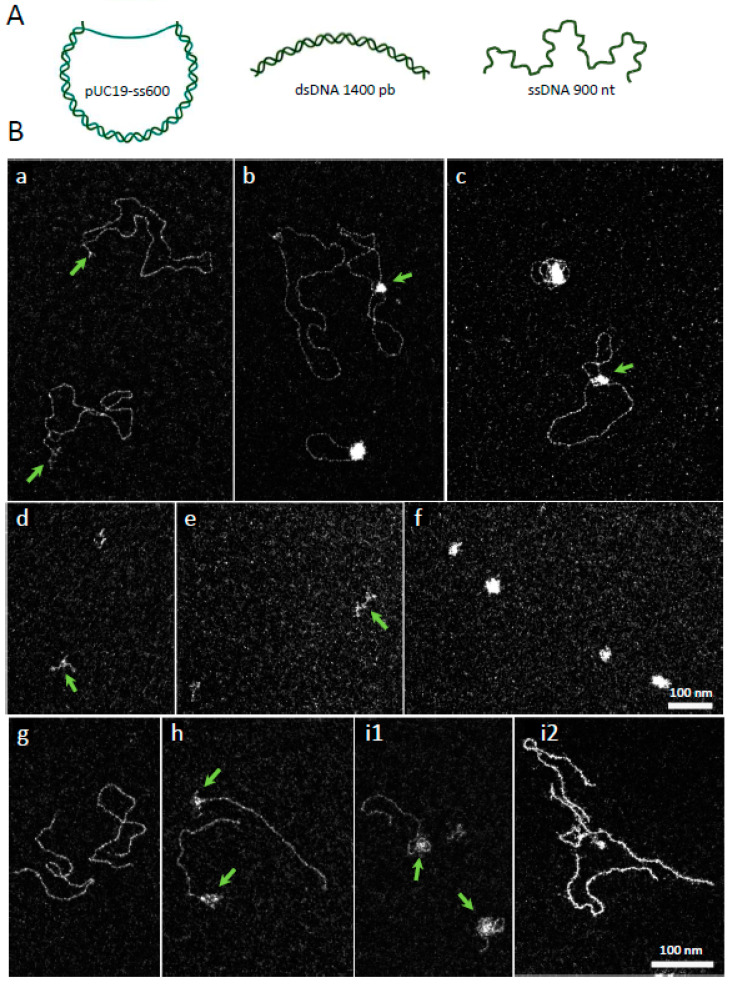
*Vc*DciA interacted preferentially on single-stranded areas of DNA or at the ss/ds junction. (**A**) Schematic representation of DNA tested for interaction with *Vc*DciA by electron microscopy. The pUC19 plasmid was modified to expose 600 unpaired nucleotides (pUC19-ss600). The linear double- and single-stranded DNA were 1440 base pairs (bp) and 900 nucleotides (nt), respectively. Created with BioRender.com (**B**) TEM images of *Vc*DciA-DNA complexes in dark-field imaging mode. (**a**) pUC19-ss600 naked DNA (control) where ssDNA is indicated by arrows. (**b**,**c**) *Vc*DciA (40 nM) first interacted with ssDNA of pUC19-ss600 (arrows) and then invaded the dsDNA. Length of free dsDNA decreased. (**d**–**f**) Interactions of *Vc*DciA with ssDNA at 40 nM (arrow) (**e**) and 100 nM (**f**), compared to the control (arrow) (**d**). (**g**,**h**,**i1**,**i2**) Interactions of *Vc*DciA (40 nM) (arrows) (**h**) and 100 nM (arrows) (**i1**,**i2**) with dsDNA, compared to the control (**g**). *Vc*DciA is bound to DNA ends, and such interactions can propagate along the dsDNA (**i2**). Scale bars correspond to 100 nm.

*Vc*DciA seems to interact with DNA without structure specificity, since the BLI assays give the same type of response to all tested DNA. In order to further characterize its mode of interaction, we visualized the contact areas of *Vc*DciA on different larger DNA substrates, including linear single- (900 nt in average) or double-stranded (1440 pb) DNA, or circular DNA, using a pUC19 plasmid containing a single-stranded DNA gap of an average length of 600 nt (named pUC19-ss600) (Figure 2A). We analyzed the complexes formed between these three substrates and variable concentrations of *Vc*DciA by transmission electron microscopy (TEM), using the positive staining spreading method and the dark-field imaging mode (Section 3).

In the absence of *Vc*DciA, pUC19-ss600 was relaxed, with the double-stranded region expanded on the grid, while the single-stranded DNA region was flexible and thus collapsed (Figure 2(Ba), arrows). In the presence of *Vc*DciA, protein•DNA complexes can be visualized. As observed by BLI, *Vc*DciA interacted with DNA. The single-stranded gap of pUC19- ss600 appears covered by the protein, even at low concentrations of *Vc*DciA (40 nM) (Figure 2(Bb,c), arrows). *Vc*DciA also interacted with double-stranded regions adjacent to the gap, applying a local condensation of the DNA. In 50% of complexes, the length of the free dsDNA was shorter, from a few nm to 450 nm (n = 213), in place of 688 ± 44 nm (n = 145) for the control, suggesting that *Vc*DciA invaded the dsDNA region of the plasmid. As in such complexes the single stranded region is always covered, we conclude that the formation of these complexes first engages the single-stranded region then invades the double-stranded region and condenses it.

The linear ssDNA alone was folded onto the grid due to its high flexibility (Figure 2(Bd)), and the addition of *Vc*DciA condensed ssDNA (Figure 2(Be)). This behavior was accentuated as the concentration of *Vc*DciA increased (Figure 2(Bf)). The *Vc*DciA•ssDNA complex did not appear as a deployed complex, as would be observable for a canonical SSB-like protein with the single strand [16], but as a globular complex that could correspond to a compaction of the ssDNA. This interaction supports the analysis of the *Vc*DciA•DNA complex with the ssDNA zone contained in pUC19-ss600 (Figure 2(Bb,c)).

In the presence of linear dsDNA (Figure 2(Bg)), *Vc*DciA formed complexes with the DNA, exhibiting several types of configurations (Figure 2(Bh,i1,i2)). The first one corresponded to the invasive presence of *Vc*DciA at the extremities of the DNA (Figure 2(Bh,i1), arrows), with the formation of a local condensation/aggregation. Some molecules displayed *Vc*DciA along the entire linear dsDNA, associated with a stiffening of dsDNA with a configuration that was difficult to characterize. We suggest that *Vc*DciA could be located between the two strands of the DNA (Figure 2(Bi2)).

The BLI experiments (Figure 1) showed that *Vc*DciA interacts with single- as with double-stranded DNA, and these microscopy observations complement the conclusions. It appears that *Vc*DciA condenses ssDNA and seems to be able to diffuse or insert directly between paired DNA strands. These transactions could result from multiple interactions of *Vc*DciA with DNA, promoting pairings between short ssDNA segments and driving multiple secondary structures, leading to condensed structures. For linear fragments, where *Vc*DciA has propagated along the double-stranded chain, we can also envisage that *Vc*DciA is located between two Watson & Crick single-stranded segments, and, therefore, this time facing each other.

### 2.3. VcDciA Protects Double-Stranded DNA Oligonucleotide Substrates from Thermal Denaturation

**Figure 3:**
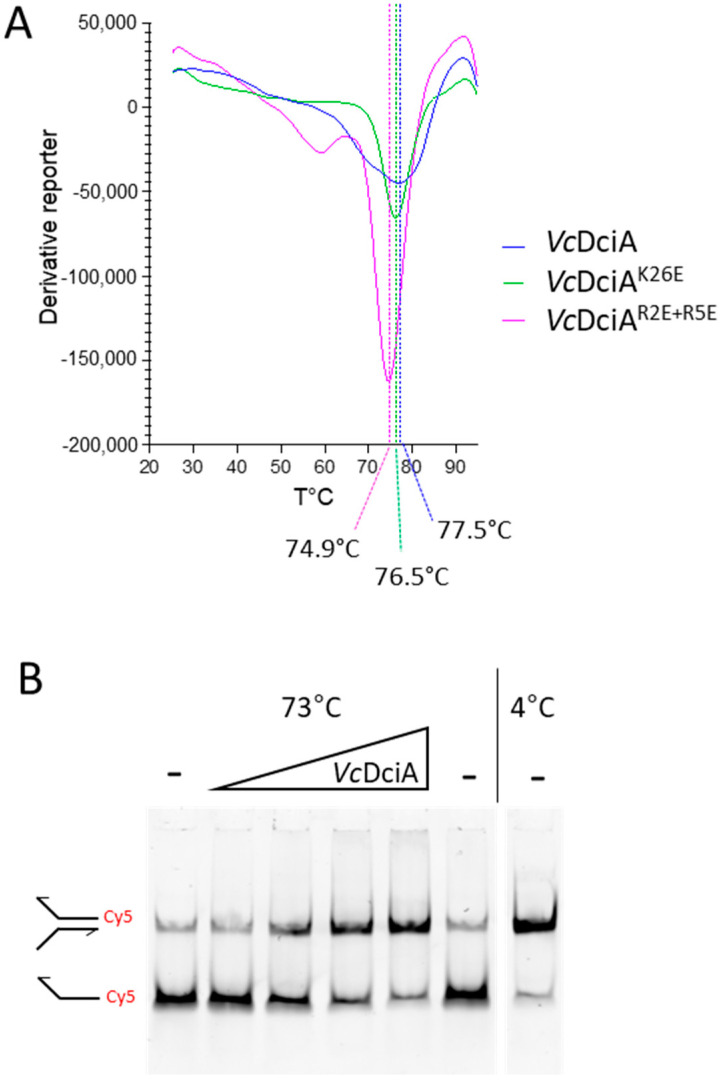
*Vc*DciA interacts with a fork-like DNA and protects it from thermal denaturation. (**A**) Thermal Shift Assays on *Vc*DciA, *Vc*DciA^R2E+R5E^, and *Vc*DciA^K26E^: first-order derivatives generated as a function of temperatures are presented. One curve of three independent experiments is presented for each protein. (**B**) The oligonucleotides forming the fork-like DNA subtract were hybridized on 25 nucleotides over 50 (nt), mimicking a replication fork (oso3 and oso4, Table 1). One of the two strands is labeled 3′ with a Cy5 fluorophore. A range of protein concentrations (0.25; 0.5; 0.75; 1 µM) was mixed with the fork-like DNA substrate and placed at 73 °C for 30 min. The DNA molecules were visualized by migration on a 6% native acrylamide gel and revealed by chemiluminescence. The controls of the forked DNA incubated at 4 °C or 73 °C without *Vc*DciA were placed on either side of the gel.

To address the hypothesis of the ability of *Vc*DciA to insert itself between the two strands of DNA, we then looked at whether DciA had an impact on dsDNA thermal stability (Figure 3). We first aimed to gain further insight into the biochemistry of *Vc*DciA by measuring its melting point, using Thermal Shift Assay (TSA) (Figure 3A). The melting point value of *Vc*DciA is high and equal to 77.5 °C. For the thermal DNA denaturation test, we used a fluorescent substrate mimicking a fork composed of the annealing of two oligonucleotides (oso3/oso4, Table 1). The double-stranded part was 25 nt long (Tm = 62.60 °C) with a 25 nt-long extension for each strand. The protein was mixed in an increasing range of concentration with a fixed amount of DNA, and the samples were heated at 73 °C for 30 min (Figure 3B). At the end of this process, the fork substrate was 80% denatured while the presence of an increasing concentration of *Vc*DciA protected the fork from thermal denaturation. Therefore, we conclude that *Vc*DciA interacts with the forked DNA and the complex stabilizes and protects the double-stranded region of the DNA.

### 2.4. The Long α-Helix 1 of VcDciA Interacts with Different DNA Substrates

In order to characterize the molecular basis of the interaction between *Vc*DciA and DNA, we continued our structural study to define the residues involved in the protein–DNA interaction.

**Figure 4:**
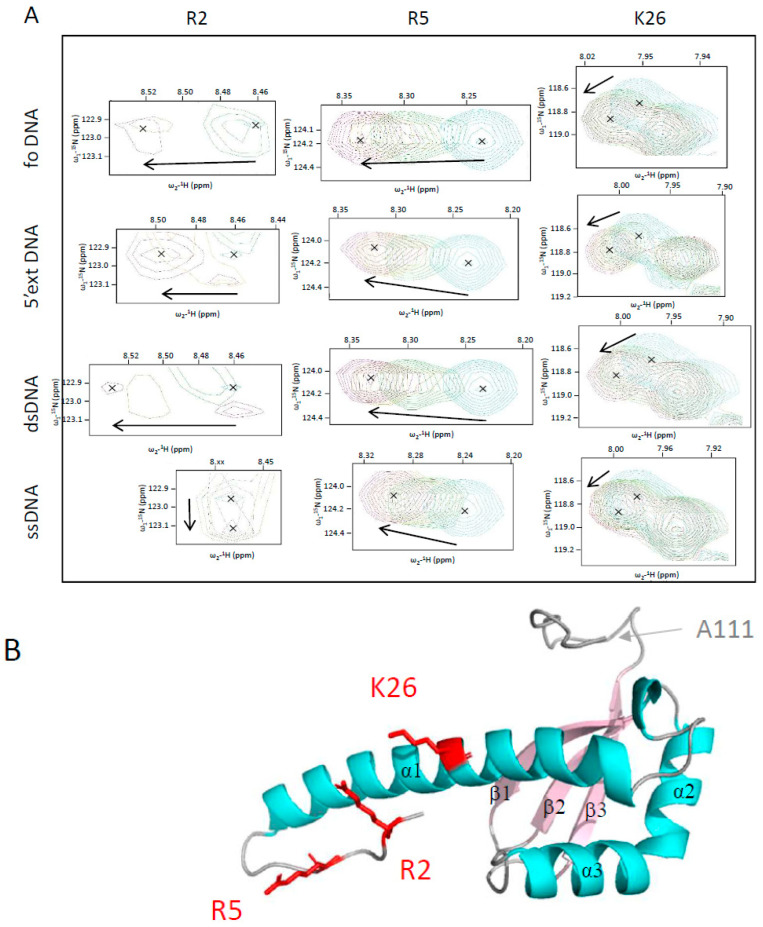
The long α-helix 1 of *Vc*DciA interacts with the four DNA substrates as identified by NMR. (**A**) Chemical shifts of R2, R5, and K26 residues of *Vc*DciA alone and after addition of DNA. Peak enlargements of the three residues were extracted from the spectra of *Vc*DciA titrated with forked (foDNA^NMR^), 5′-extDNA (free 5′ end), double-stranded (dsDNA^NMR^), and single-stranded (ssDNA^NMR^) DNA (Table 1). Sofast-HMQC spectra were recorded at protein–DNA ratios of 1:0, 1:0.5, 1:1, and 1:2 molar ratios (cyan, yellow, red, and purple, respectively). Increasing the amount of DNA caused the peaks of the 3 residues to shift. The centers of the peaks are marked with X, and an arrow marks their shifts. The interaction of R2 with DNA also induced signal attenuation. (**B**) Schematic representation of *Vc*DciA^[1–111]^. The α-helices are in cyan and the β-strands are in pink. Residues selected to generate mutated versions of *Vc*DciA are indicated on the structure of *Vc*DciA^[1–111]^. The lateral chains of the R2, R5, and K26 residues are shown in red sticks. They were mutated in aspartic acids in order to validate their impact on *Vc*DciA binding to DNA and on the helicase loading.

The CTD-deleted version of *Vc*DciA, corresponding to the M1 to A111 residues, was uniformly labeled with ^15^N in order to map the DNA interaction areas on the KH-fold domain of *Vc*DciA using NMR spectroscopy. The assignment of the ^1^H-^15^N correlation spectrum was taken from our previous structural study of *Vc*DciA (Supplementary Figure S3) [7]. Four small DNA substrates were added to the labeled protein: the mimicking fork foDNA, a dsDNA (18 base pairs), a ssDNA (18 nucleotides), and a dsDNA extended 5′ (9 nucleotides, 5′ext DNA) (Table 1). For the four tested DNA substrates, we observed chemical shift variations as well as changes in the intensities of signals (Supplementary Figure S4). Few ^15^N-*Vc*DciA^[1–111]^ signals showed an important decrease in intensity in the presence of DNA (Supplementary Figure S5A–H left, red and yellow bars). In contrast, positively charged residues located in the α-helix 1 (R2, R5, and K26) of the protein showed significant chemical shift changes upon the addition of the four DNA substrates, with a rapid exchange regime as exemplified in Figure 4A (and red bars in Supplementary Figure S5A–H right). The fact that α-helix 1 was involved in the interaction with DNA was atypical compared to the known mode of interaction of KH domains with nucleic acids [14,17]. These results confirm that *Vc*DciA does not interact with a specific DNA structure and support the hypothesis that DciA could intercalate between the two DNA strands, perhaps via its large α-helix 1. Only the ssDNA shows a little less impact in this zone of *Vc*DciA.

### 2.5. The R2, R5, and K26 Residues Involved in the Interaction of VcDciA with the DNA Have a Different Impact on the Helicase Loading

**Figure 5:**
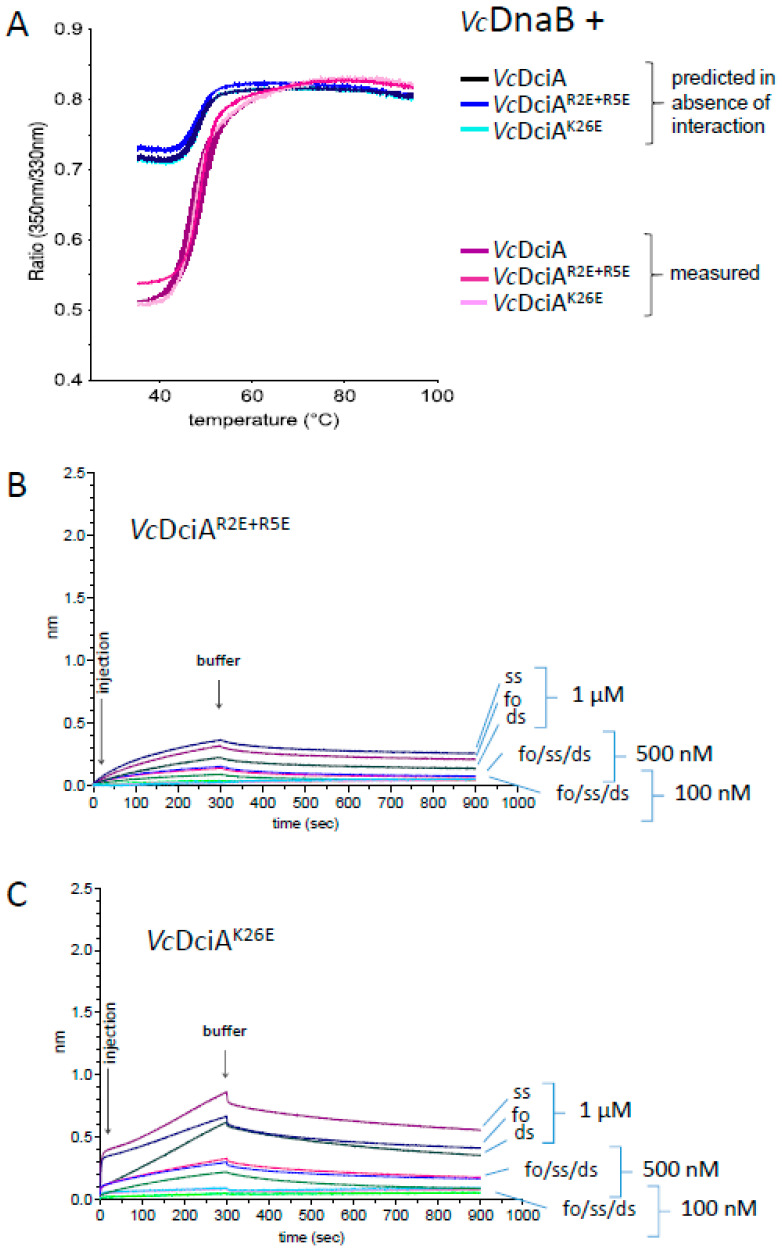
Biochemical validation of the R2, R5, and K26 residues for their interaction with the DNA. (**A**) Characterization of the interaction between *Vc*DnaB and *Vc*DciA, and *Vc*DciA^R2E+R5E^ and *Vc*DciA^K26E^ by Tycho NT.6 analysis. In 3 shades of pink are reported the 350/330 nm ratio obtained for the 3 complexes and in 3 shades of blue are reported the predicted ratio for an absence of solvent protection. The curves correspond to the mean ± SEM of three analyses. The interactions of *Vc*DciA^R2E+R5E^ (**B**) and *Vc*DciA^K26E^ (**C**) with single- and double-stranded DNA, and with a fork-mimicking DNA, using BLI technology, are observed as for *Vc*DciA (Figure 1).

In order to focus our study on the area of *Vc*DciA most impacted by the interaction with the DNA, three basic residues, R2, R5, and K26 were mutated to glutamic acids, in order to reverse the charge while minimizing disruption of the steric hindrance of the side chain. R2 and R5 are located at the extremity of α-helix 1 while K26 is located in the middle (Figure 4B). The double mutant R2E+R5E and the single mutant K26E were expressed and purified according to the same protocol as for *Vc*DciA (Section 3). Their overall stability, which reflects their correct folding, was estimated by measuring their denaturation temperature using TSA, which is high and close to that of the wild-type protein, with an apparent Tm of 74.9 and 76.5 °C for *Vc*DciA^R2E+R5E^ and *Vc*DciA^K26E^, respectively, to be compared to the apparent Tm of 77.5 °C for *Vc*DciA (Figure 3A). To verify their structural integrity, their ability to interact with *Vc*DnaB was monitored and confirmed by the DSF technique, which was used previously (Figure 5A) [7].

The interaction of the two *Vc*DciA mutants with DNA was tested by BLI with the same three DNA substrates as for *Vc*DciA (see Figure 5B,C, compared with Figure 1). For both mutants, *Vc*DciA^R2E+R5E^ and *Vc*DciA^K26E^, the response intensity profiles were much lower, indicating that the mutations strongly affected the protein-DNA interaction, as expected from the NMR analysis. A decrease in the interaction signal was observed for all DNA substrates, especially for the *Vc*DciA^R2E+R5E^ double mutant, and less so for *Vc*DciA^K26E^.

**Figure 6:**
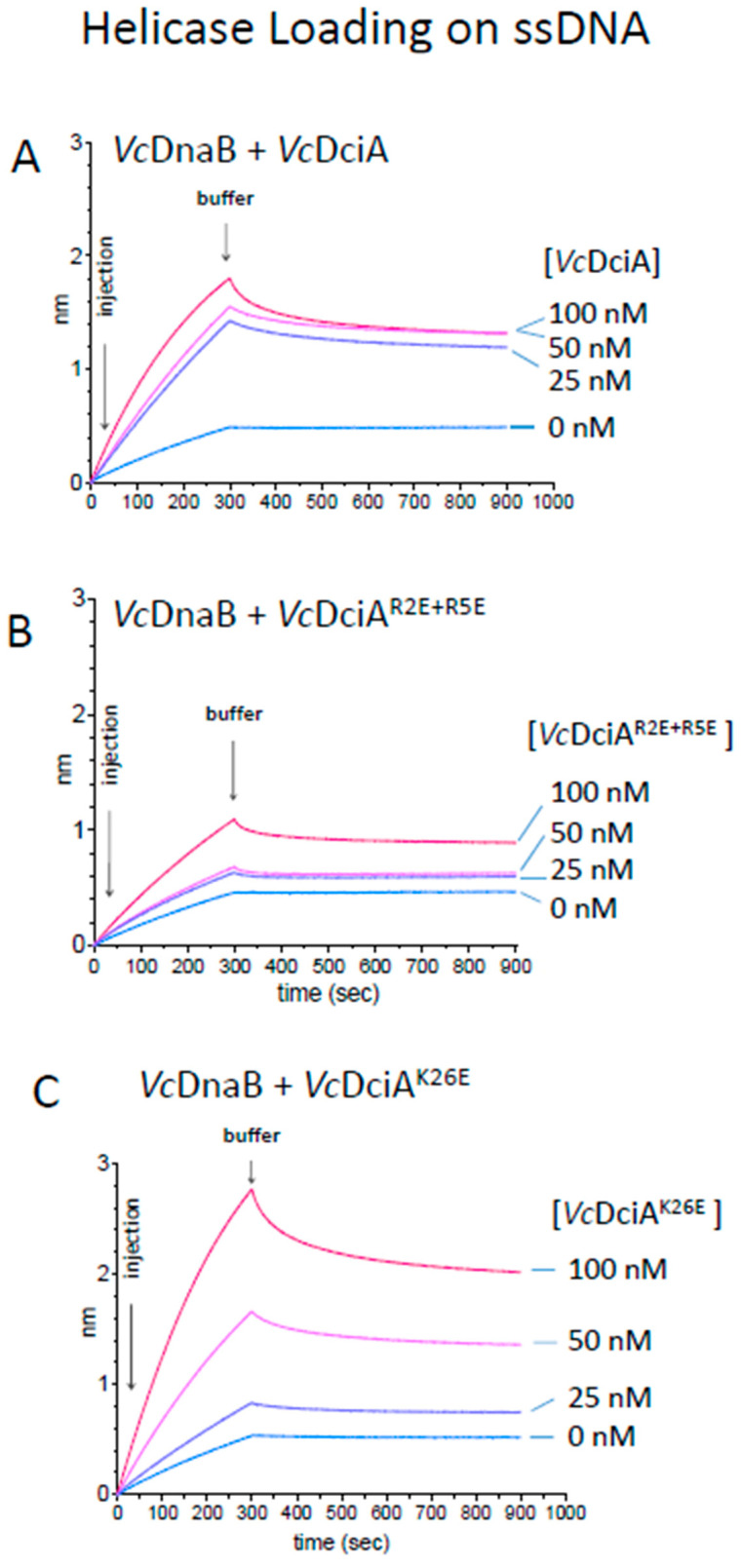
*Vc*DnaB loading by *Vc*DciA^R2E+R5E^ and *Vc*DciA^K26E^ compared to *Vc*DciA. Bio-layer interferometry (BLI) analysis using biotinylated oligonucleotide oso23 (80 nt) immobilized onto the surface of a streptavidin (SA)-coated probe by its 3′ extremity (see Section 3). Association was performed with the *Vc*DnaB helicase at a concentration of 100 nM during 300 s in a buffer solution containing ATP. Dissociation was assessed in the same buffer for 600 s. Increasing loader concentrations (0, 25, 50, and 100 nM in subunits, in cyan, blue, pink, and red, respectively) were analyzed. (**A**) *Vc*DnaB loading to ssDNA in the presence of *Vc*DciA. (**B**) *Vc*DnaB loading to ssDNA in the presence of *Vc*DciA^R2E+R5E^. (**C**) *Vc*DnaB loading to ssDNA in the presence of *Vc*DciA^K26E^.

The impact of these mutations on the helicase loading was finally tested on the different DNA substrates according to the same protocol using BLI technology described previously [9] (Figure 6 and Supplementary Figures S6 and S7). We tested the loading of *Vc*DnaB (100 nM) in the presence of three different concentrations of *Vc*DciA (25, 50, and 100 nM). For all substrates, we observed that at the highest concentration of *Vc*DciA (100 nM), the signal of the three DciA in the absence of *Vc*DnaB was negligible (Figure 1 and Figure 5B,C), indicating that the observed signal was due to the *Vc*DnaB loading.

We first observed the *Vc*DnaB loading on the ssDNA oligonucleotide (Figure 6). As published previously, *Vc*DciA increases the loading of *Vc*DnaB, and the maximum loading is obtained as of the lowest tested concentration (Figure 6A) [7]. For *Vc*DciA^R2E+R5E^, the efficiency in the *Vc*DnaB loading decreased strongly (Figure 6B), as observed for its DNA binding property (Figure 5B), which suggests that the two functions may be related. For *Vc*DciA^K26E^, however, the loading of *Vc*DnaB was surprisingly very efficient and increased proportionally to the concentration of DciA mutant (Figure 6C). It is difficult, for the moment, to explain this result, knowing that the mutated protein harbors a high deficiency in DNA binding compared to *Vc*DciA (compare Figure 5C with Figure 1).

Finally, we tested the loading of *Vc*DnaB on the foDNA and the dsDNA substrates that were used in the *Vc*DciA binding studies (Supplementary Figures S6 and S7, respectively). For foDNA, the results were comparable to those obtained with the ssDNA substrate (compare Supplementary Figure S6 to Figure 6). For dsDNA, whereas SSB interaction was very low (Supplementary Figure S1), confirming the absence of single-stranded DNA, we observed a slight loading of *Vc*DnaB, alone and stimulated by *Vc*DciA (Supplementary Figure S7A). Again, as for ssDNA and foDNA, the stimulation was very low with *Vc*DciA^R2E+R5E^ (Supplementary Figure S7B), but it was maintained, or perhaps even slightly increased, by *Vc*DciA^K26E^ (Supplementary Figure S7C).

## 3. Materials and Methods

### 3.1. Protein Samples Preparation and Site-Directed Mutagenesis

*Vc*DciA, *Vc*DciA^R2E+R5E^, and *Vc*DciA^K26E^ were all 6His-tagged at the N-terminus during the cloning process and were over-expressed in the *E. coli* Rosetta(DE3)pLysS strain and purified as described in [7] in two steps: Ni-NTA and ion exchange on heparin (pH 5.6). The mutants were constructed by site-directed mutagenesis using non-overlapping and 5′-phosphorylated oligonucleotides (Eurofins, Luxembourg), which introduced the desired changes. The oligonucleotides used were the following: mutant *Vc*DciA^R2E+R5E^ 5′-CACGAGGATCACGAACCTACCGCT-3′ and 5′-ATGGTGATGGTGATGCATATGTAT-3′; and mutant *Vc*DciA^K26E^ 5′-GAGCATGCAGAAGCGATTTTGC-3′ and 5′-TTGGATCTGCTTGAGTTTGGATGC-3′. Using the pET29-*Vc*DciA plasmid as a template, the complete plasmids were amplified by PCR in a linear product and circularized by ligation. The introduction of mutations was verified by sequencing (Genewiz, Azenta Life Sciences, New Brunswick, NJ, USA). NTD-6His-tagged *Dr*SSB (*Deinococcus radiodurans*) was purified by a first step on Ni-NTA in 20 mM Tris-HCl (pH 7.5) + 1 M NaCl, followed by a final chromatographic step on a Superdex column in 20 mM Tris-HCl (pH 7.5) + 200 mM NaCl, in order to complete the purification. 

### 3.2. Measurement of VcDciA–DNA Interaction by Bio-Layer Interferometry (BLI)

The interaction experiments of *Vc*DciA with DNA by BLI were conducted using a Sartorius Octet^®^ RED96e system (Fremont, CA, USA) and Streptavidin (SA) Biosensors. Three different substrates were immobilized on the SA sensors, and their interactions were compared. The ssDNA corresponded to the 80 nt long oso23 harboring a biotin on its 5′ extremity (Table 1). The foDNA mimics a DNA fork and was obtained by the annealing of oso23 with oso18, presenting a hybridized zone of 38 nucleotides. The dsDNA was obtained by the annealing of oso23 with oso24. Each substrate at 40 nM was immobilized in HN buffer (50 mM Hepes (pH 7.0) + 150 mM NaCl) onto the surface of the SA biosensor through a cycle of Baseline (120 s), Loading (120 s), and Baseline (120 s), in order to obtain 0.5 nm of signal. After immobilization of DNA on the sensor, association was performed during 300 s in wells containing 200 µL samples at 0.1, 0.5, and 1 µM of *Vc*DciA, *Vc*DciA^R2E+R5E^, and *Vc*DciA^K26E^ in buffer HNTA (HN buffer with Tween 0.1% and 1 mM ATP). At the end of each binding step, the sensors were transferred into a protein-free binding buffer HNTA to follow the dissociation kinetics for 600 s. The sensors can be recycled by dipping in TNT buffer (100 mM Tris-HCl (pH 8.8) + 1 M NaCl + Tween 0.1%). The experiments were carried out in duplicate; only one is presented. For each sensorgram presented, the non-specific contribution obtained on a bare sensor was subtracted from the functionalized sensor. The integrity of the DNA substrates was verified under the same experimental conditions by using the SSB protein at 300 mM, which only binds to ssDNA.

### 3.3. Electron Microscopy

The positive staining method and dark-field imaging mode were used to analyze *Vc*DciA- DNA complexes [18]. Hexagonal 600 mesh copper grids, previously covered with a thin carbon film, were functionalized in a homemade device by glow-discharge in the presence of amylamine, providing NH^3+^ charge deposition onto the carbon surface. This allowed gentle adsorption of negatively charged DNA. The complexes were reconstituted after a 30 min incubation at 4 °C by mixing 40 or 100 nM of *Vc*DciA with pUC19-ss600, and dsDNA or ssDNA at a concentration of 3 nM molecules each, in 10 mM Tris-HCl buffer (pH 7.5) + 120 mM NaCl + 1 mM ATP + 5 mM Mg^2+^. Five μL of complexes (0.25 μg/mL DNA) were deposited on the activated carbon film for 1 min and then rinsed with aqueous 2% uranyl acetate and dried to both stain the sample and spread it on the surface. The samples were observed in dark-field imaging mode and by filtering the electrons in order to keep the electrons that had not lost energy (so-called “Zero Loss Mode”), thanks to an omega energy loss filter included in the column of the transmission electron microscope model Zeiss 912AB (120 kV). The images were captured with a Tengra CCD camera at magnifications from 20,000 to 63,000 and analyzed by Item software (both Olympus (Tokyo, Japan), Soft Imaging Solutions).

### 3.4. Measurement of the Melting Point of VcDciA and of the VcDciA^R2E+R5E^ and VcDciA^K26E^ Mutants by Thermal Shift Assay (TSA)

The thermostability of *Vc*DciA, *Vc*DciA^R2E+R5E^, and *Vc*DciA^K26E^ was assessed with a StepOnePlus Real Time PCR system (Applied Biosystems). Sypro Orange fluorescent dye, which non-specifically binds to hydrophobic regions, was used to measure thermal denaturation of the protein at 488 nm during the thermal ramp, starting from 25 °C up to 95 °C, with a scan rate of 1 °C per minute. The assays were performed in duplicates or triplicates in 96 well plates in a final volume of 30 µL, with a protein concentration of 0.5 mg/mL and Sypro Orange of 5× in HN buffer (50 mM Hepes (pH 7.0) + 150 mM NaCl).

### 3.5. Protection of the dsDNA from Thermal Denaturation by VcDciA

The ability of *Vc*DciA to protect a replication fork mimicking DNA from thermal denaturation was studied after pairing oso3 and oso4 DNA (Table 1) in hybridization buffer (60 mM Hepes (pH 7.0) + 180 mM NaCl + 10 mM MgCl_2_). The oso3 strand was labeled at the 5′-end with a Cy5 fluorophore. The Tm of the hybridized zone was 62.60 °C (https://dna-polymerase.com/tm-calculator, accessed on 5 January 2023). *Vc*DciA was mixed at 0.25; 0.5; 0.75, or 1 µM final with 4 nM of the prepared forked DNA in 10 µL final. The samples were placed in a dry bath at 73 °C for 30 min. Reactions were stopped on ice, and 0.5% SDS (final concentration) was added before loading on a 10% acrylamide gel in TBE 1X. The DNA controls without DciA consisted of the same 30 min incubation at 73 °C, and at 4 °C. The results were revealed by using a Typhoon imager (Cytiva).

### 3.6. NMR Experiments

The ^15^N-*Vc*DciA^[1–111]^ protein was produced with a minimal media expression protocol as in [7], and the same purification protocol as *Vc*DciA^[1–111]^ was applied. The protein was concentrated at 10 mg/mL in 300 µL (544.43 µM) in the final buffer composed of 20 mM Phosphate (pH 5.6) + 1.3 M NaCl. For NMR assays, ^15^N-*Vc*DciA^[1–111]^ was diluted in 500 µL in 20 mM Phosphate Buffer (pH 5.6) + 50 mM NaCl to reach the concentration of 26 µM. A titration at 303 K at protein–DNA molar ratios of 1:0.5, 1:1, and 1:2 was then performed with four DNA substrates: the foDNA mimicking a fork, a dsDNA (18 base pairs), a ssDNA (18 nucleotides), and a dsDNA with a 9-nucleotide long free 5′ end (5′ext DNA) (Table 1). At each titration point, the sofast-HMQC ^1^H-^15^N spectrum was acquired on a 700 MHz spectrometer with a cryoprobe, at 303 K. Spectra with or without the addition of the highest concentration of DNA were also recorded at 293 K.

Intensity variations I/I^0^ were measured for each of the ^15^N-*Vc*DciA^[1–111]^ signals (I^0^ referring to the intensity of the free protein). The results are presented at the protein–DNA molar ratio 1:2 for the two temperatures (293 K and 303 K) (Supplementary Figure S5, left panels).

Chemical shift variations were calculated using the following formula: $\sqrt{{\left(\delta_{HN}^{}-\delta_{HN}^{0}\right)}^{2}+{\left(0.17\left(\delta_{N}^{}-\delta_{N}^{0}\right)\right)}^{2}}$, where *δ* represents the measured chemical shift value. Superscript 0 refers to the free form of the protein, HN or N for the amide proton or nitrogen, respectively. The factor 0.17 corresponds to the scaling factors used to normalize the magnitude of the proton and nitrogen chemical shift changes (in ppm) [19] (Supplementary Figure S5, right panels).

### 3.7. Protein–Protein Interaction Analysis by Thermal Shift Assay and Intrinsic Fluorescence Variation (DSF Differential Scanning Fluorescence)

As described in [7], intrinsic fluorescence changes in tryptophan (and tyrosine at a lower level) were recorded at 330 and 350 nm while heating the protein sample from 35 to 95 °C at a rate of 3 °C/min. The emission profile of the tryptophan was shifted to the red emissions when it was released to the solvent during the thermal denaturing of the protein. We used DSF analysis (Tycho NT.6, NanoTemper Technologies GmbH, Munich, Germany) to follow the interaction between *Vc*DnaB and *Vc*DciA, *Vc*DciA^R2E+R5E^, or *Vc*DciA^K26E^. Interaction experiments were performed in 50 mM Hepes (pH 7.5) + 150 mM NaCl + 1 mM ATP, with 20 µM of each protein, in glass capillaries of 10 µL. Three to five replicates were obtained to increase confidence in the results. To detect binding, we compared the 350/330 nm ratio of fluorescence of the complex with the predicted ratio we should obtain in the absence of interaction considering the fluorescence additivity of the proteins alone (S brightness at 350 nm/S brightness at 330 nm).

### 3.8. Measurement of the Activation of VcDnaB Loading by VcDciA or Mutants by Interaction by Bio-Layer Interferometry (BLI)

The loading of the *Vc*DnaB helicase in the presence of *Vc*DciA, *Vc*DciA^R2E+R5E^, and *Vc*DciA^K26E^ was monitored by BLI on the oso23 substrate immobilized on the sensors, as described previously in Section 3.2. The loading experiments were performed as described previously [9]. Briefly, association interactions were monitored during 300 s in wells containing 200 µL samples at 100 nM of *Vc*DnaB with different ratios of the indicated loader in buffer HNTA (50 mM Hepes pH 7, 150 mM NaCl, 1 mM ATP, 0.1% Tween20). At the end of each binding step, the sensors were transferred into a protein-free binding buffer HNTA to follow the dissociation kinetics for 600 s. The sensors can be recycled by dipping in TNT buffer (100 mM Tris-HCl (pH 8.8) + 1 M NaCl + Tween 0.1%).

## 4. Conclusions

In addition to being able to interact with single- and double-stranded DNA, *Vc*DciA can bind to forked DNA, but in an atypical way for a KH domain, and it can maintain the integrity of that DNA under exposure to high temperatures. Positive staining electron microscopy experiments also show that *Vc*DciA appears to target double-to-single-stranded DNA transition zones and then folds or condenses the DNA, perhaps by intercalating between the two DNA strands. *Vc*DciA would, therefore, have DNA stabilizing properties. We thus propose that *Vc*DciA could play a role as a DNA chaperone protein, favoring the most favorable thermodynamic states for the complexes. The results obtained with the mutated protein *Vc*DciA^R2E+R5E^ are in agreement with this hypothesis, since both DNA affinity and helicase loading were affected. However, the K26E mutation results in an intriguing protein change. Indeed, the *Vc*DciA^K26E^ interaction with DNA is strongly reduced, whereas the *Vc*DnaB loading activity is conserved, indicating that DciA DNA binding is not the only decisive function for helicase loading. A detailed analysis of the entire NMR data should provide more information to understand how DciA functions in the important process of the initiation of DNA replication in bacteria. It is known that during replication, ssDNA is bound to SSB in order to be protected. It is therefore difficult to imagine that DciA could interact with ssDNA without having first removed SSB, as DprA does, for example. However, our experiments tend to show that DciA is able to bind to ssDNA in a dsDNA context, which is not the case for the SSB protein. Future investigations to understand the mode of action of DciA on DNA and on the helicase will have to focus on this property of DciA, which involves the α-helix 1 but probably also other regions of DciA.

## Figures and Tables

**Table 1:** Sequences of the oligonucleotides used in BLI and NMR analyses.

Name	Size (nt)	Sequence (5′→3′)
oso3	50	*Cy5*-GCAGGCTCGTTACGTAGCTGTACCG-dT(25)
oso4	50	dT(25)-CGGTACAGCTACGTAACGAGCCTGC
oso18	88	CCAGGAATACGGCAAGTTGGAGGCCGGGCTGGATGGAGACTAAGCTTTGGAAGTGAAGGTTTCGAATCAGAGGTAGGTTTCACCACGC
oso23	80	*Biotin*-AAGCGTGGTGAAACCTACCTCTGATTCGAAACCTTCACTTTACGTGGTCTGGCGTGGTGAATGTTCGTCGGCGTGCTCGA
oso24	78	TCGAGCACGCCGACGAACATTCACCACGCCAGACCACGTAAAGTGAAGGTTTCGAATCAGAGGTAGGTTTCACCACGC
foDNA^NMR^	18/18	GCTGTACCGTTTTTTTTT and TTTTTTTTTCGGTACAGC
dsDNA^NMR^	18	GCTGTACCGACTAGCAAT and ATTGCTAGTCGGTACAGC
ssDNA^NMR^	18	GCTGTACCGACTAGCAAT
5′extDNA^NMR^	18/9	TTTTTTTTTCGGTACAGC and GCTGTACCG

## Data Availability

The data presented in this study are available on request from the corresponding authors.

## Supplementary Materials

The following supporting information can be downloaded at: https://www.mdpi.com/article/10.3390/ijms24021427/s1.
