# Supplementary material for: Structural Insights of the DciA Helicase Loader in Its Relationship with DNA
Source: Int J Mol Sci. 2023 Jan 11;24(2):1427. doi: 10.3390/ijms24021427 (PMC9865707; doi:10.3390/ijms24021427)

Structural insights of the DciA helicase loader in its relationship with DNA

Claire Cargemel[1], Sonia Baconnais[2], Magali Aumont-Nicaise[1], Magali Noiray[1], Lia Maurin[1], Jessica Andreani[1], Hélène Walbott[1], Éric Le Cam[2], Françoise Ochsenbein[1], Stéphanie Marsin[1*], Sophie Quevillon-Cheruel[1*]

Supplementary Figure Legends

Supplementary Figure S1. Validation of the DNA substrates for BLI experiments by *Dr*SSB. The *Dr*SSB protein was used to control the integrity of the three DNA substrates. The experiment is conducted as for *Vc*DciA in Figure 1.

Supplementary Figure S2. The *Vc*DciA/DNA binding curves from Figure 1 were reproduced and each sensorgram (shown in solid lines) was fitted (dotted lines) using a 1:1 (Langmuir) binding model in Octet data analysis software. The differences between dotted and solid lines indicates that the interactions do not follow the 1:1 binding model.

Supplementary Figure S3. Assigned ^{1}H-^{15}N Sofast-HMQC spectrum of ^{15}N-*Vc*DciA$^{[1-111]}$ at 303°K, derived from previous assignment at 293°K [7]. Unassigned peaks correspond to the side chains of Asn and Gln residues (upper right region).

Supplementary Figure S4. Superimposed ^{1}H-^{15}N Sofast-HMQC spectra of ^{15}N-*Vc*DciA$^{[1-111]}$ alone (cyan) and after addition of different DNA at increasing concentrations (fork, 5'-end, double or single stranded DNA) corresponding to the molar ratios 1:0.5 (green), 1:1 (yellow), 1:2 (magenta). Spectra were recorded at 293K and 303K and at 700 MHz. Schematics of the DNAs used are shown at the left side.

Supplementary Figure S5. Variations in $^{1}H^{15}N$ Sofast-HMQC signal intensities (ratio I/I_0, left) and chemical shifts perturbations ($\Delta\delta^{1}H^{15}N$, right) of *Vc*DciA$^{[1-111]}$ residues upon addition of the DNA substrates at 293K **(A, B, C, D)** and 303K **(E, F, G, H)**. Highly impacted residues are

indicated in red, moderately perturbated signals in yellow, and no significant perturbation is indicated in green.

Supplementary Figure S6. *Vc*DnaB loading by *Vc*DciA$^{R2E+R5E}$ and *Vc*DciA^{K26E} compared to *Vc*DciA, on a fork DNA substrate. Bio-layer interferometry (BLI) analysis using biotinylated oligonucleotide oso23 hybridized with oso18 (Table1) to form the foDNA substrate immobilized onto the sensor (see Section 3). Association was performed with the *Vc*DnaB helicase at a concentration of 100 nM during 300 s in a buffer solution containing ATP. Dissociation was assessed in the same buffer for 600 s. Increasing loader concentrations (0, 25, 50 and 100 nM in subunits, in cyan, blue, pink and red respectively) were analyzed. **(A)** *Vc*DnaB binding on foDNA in the presence of *Vc*DciA. **(B)** *Vc*DnaB binding on foDNA in the presence of *Vc*DciA$^{R2E+R5E}$. **(C)** *Vc*DnaB binding on foDNA in the presence of *Vc*DciA^{K26E}.

Supplementary Figure S7. DnaB loading by *Vc*DciA$^{R2E+R5E}$ and *Vc*DciA^{K26E} compared to *Vc*DciA, on a dsDNA substrate. Bio-layer interferometry (BLI) analysis using biotinylated oligonucleotide oso23 (80nt) hybridized with oso24 to form the dsDNA substrate immobilized onto the sensor (see Section 3). Association was performed with the *Vc*DnaB helicase at a concentration of 100 nM during 300 s in a buffer solution containing ATP. Dissociation was assessed in the same buffer for 600 s. Increasing loader concentrations (0, 25, 50 and 100 nM in subunits, in cyan, blue, pink and red respectively) were analyzed. **(A)** *Vc*DnaB binding on dsDNA in the presence of *Vc*DciA. **(B)** *Vc*DnaB binding on dsDNA in the presence of *Vc*DciA$^{R2E+R5E}$. **(C)** *Vc*DnaB binding on dsDNA in the presence of *Vc*DciA^{K26E}.

Supplementary Figure S1

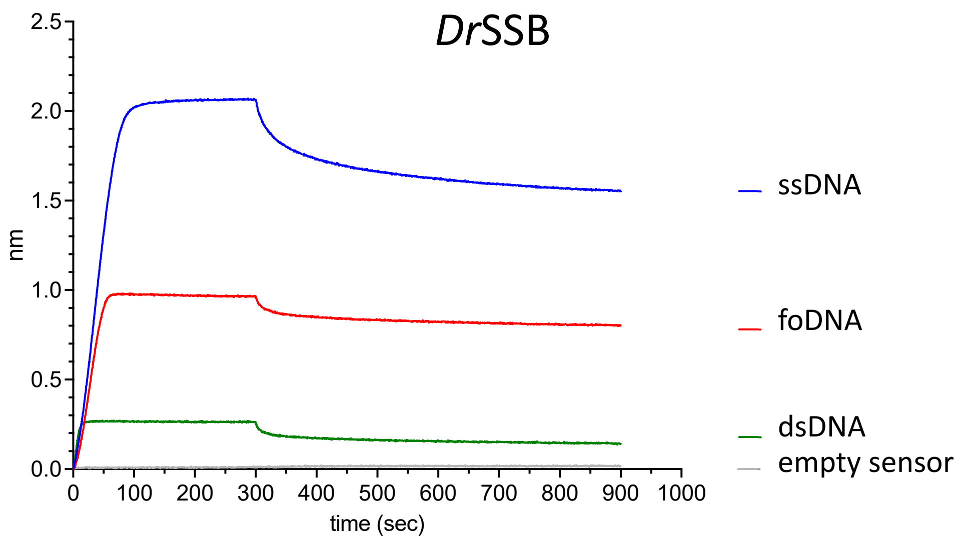

Supplementary Figure S2

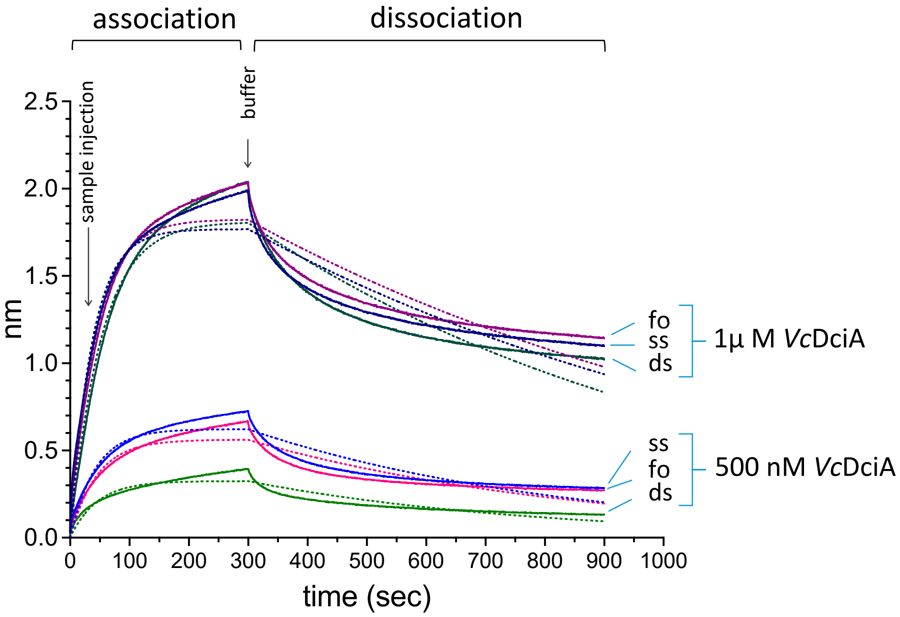

Supplementary Figure S3

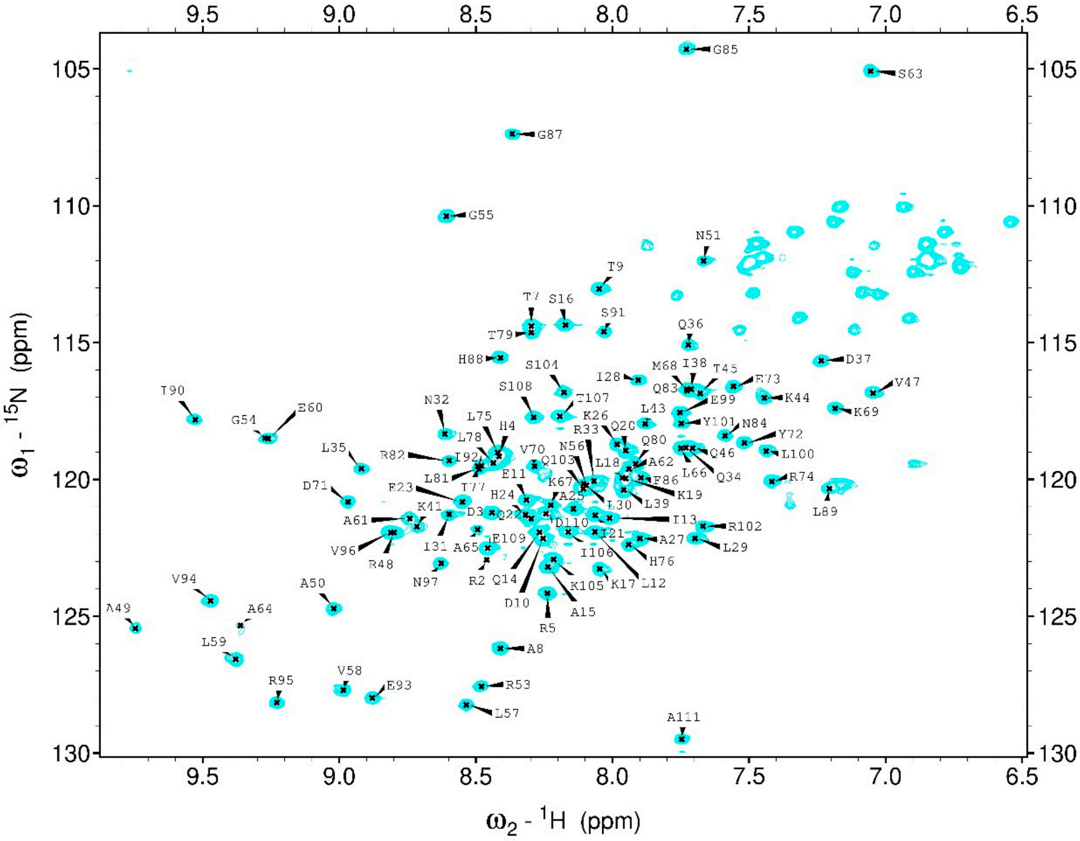

Supplementary Figure S4

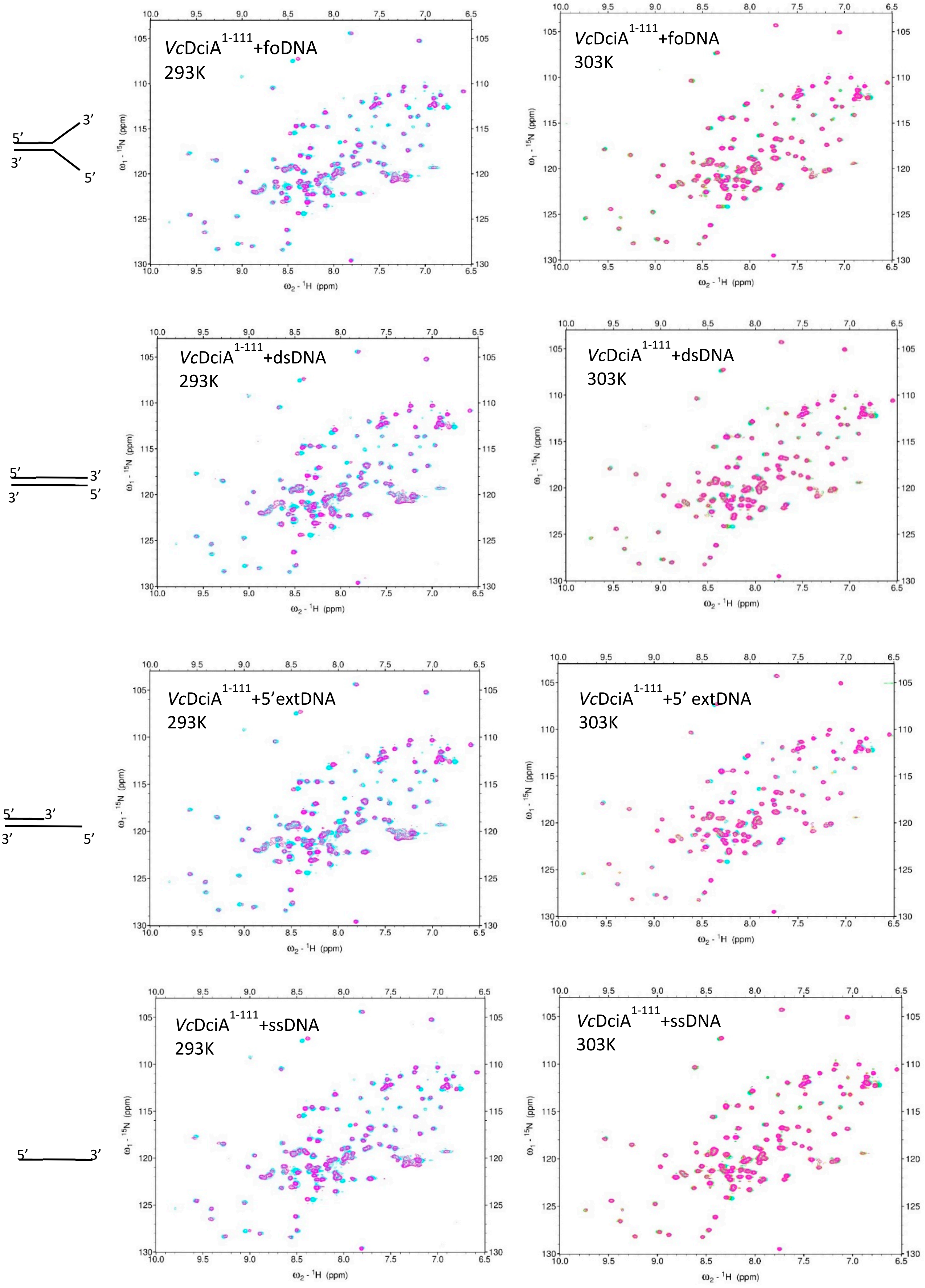

Supplementary Figure S5

293 K

A *Vc*DciA1-111 + foDNA

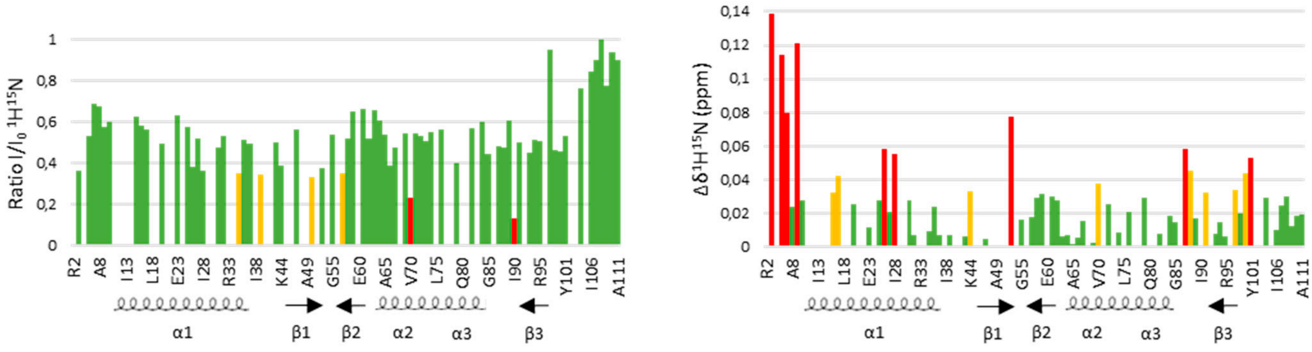

B *Vc*DciA1-111 + 5'-ext DNA

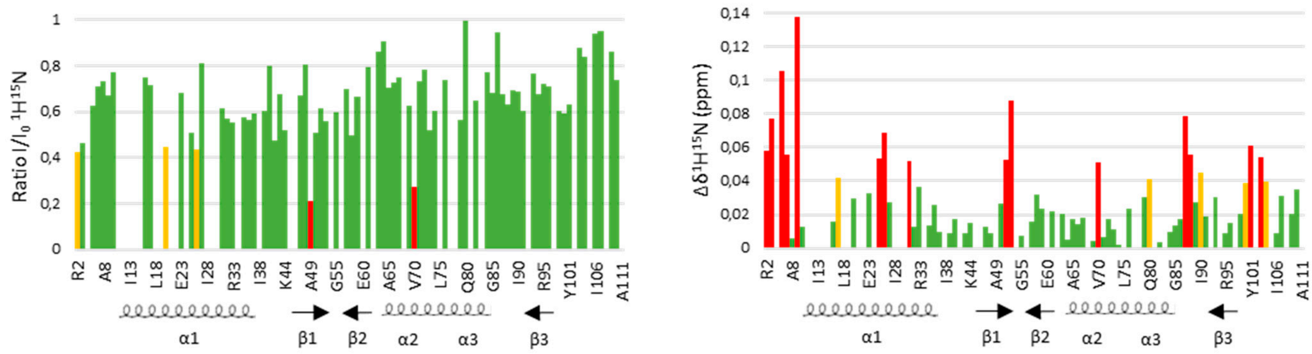

C *Vc*DciA1-111 + dsDNA

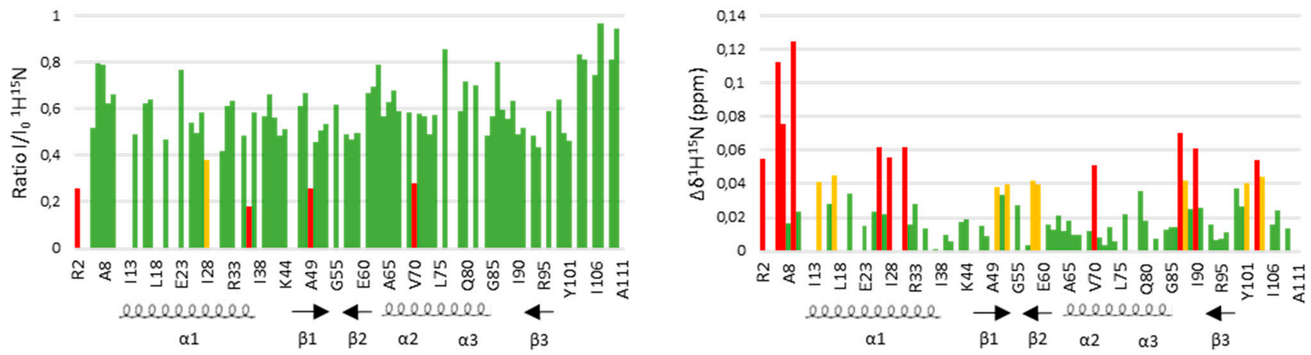

D *Vc*DciA1-111 + ssDNA

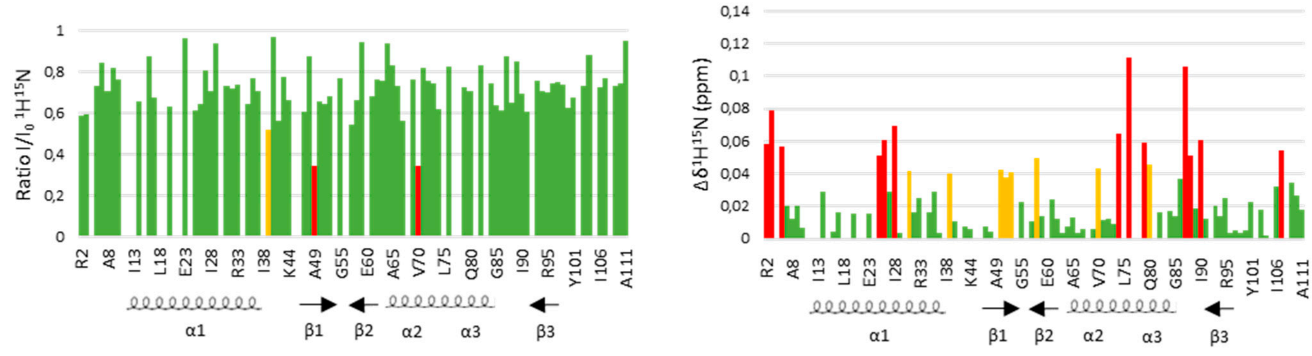

Supplementary Figure S5 (end)

303 K

E *Vc*DciA$^{1-111}$ + foDNA

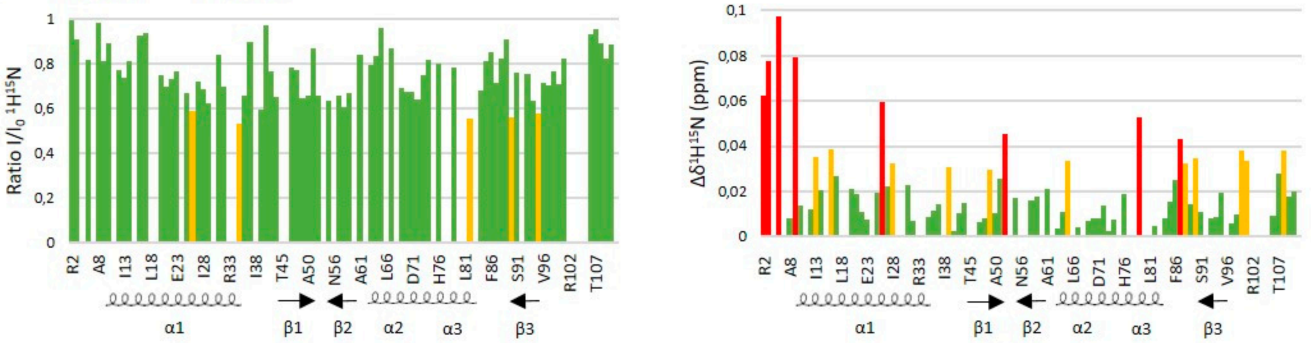

F *Vc*DciA$^{1-111}$ + 5'-ext DNA

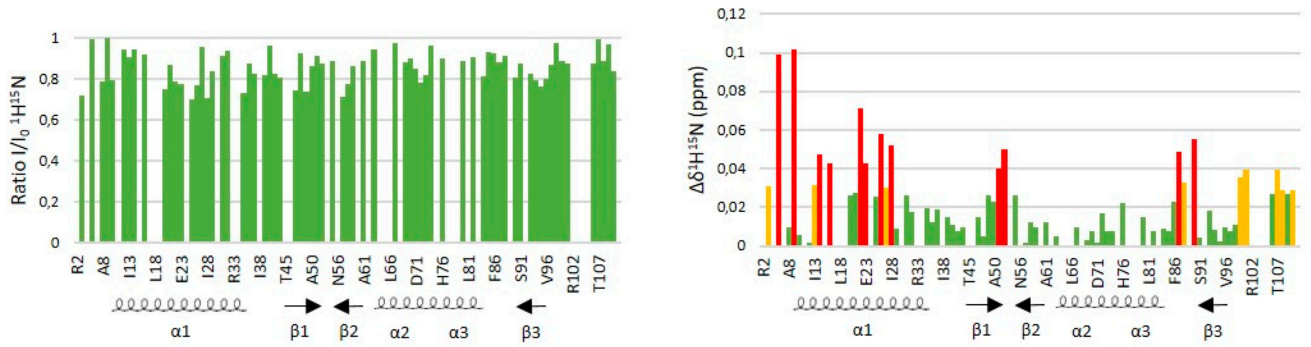

G *Vc*DciA$^{1-111}$ + dsDNA

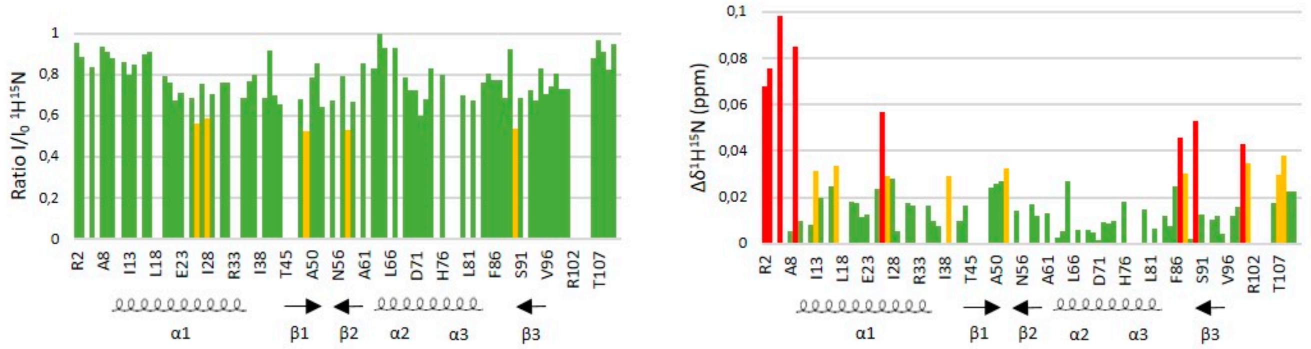

H *Vc*DciA$^{1-111}$ + ssDNA

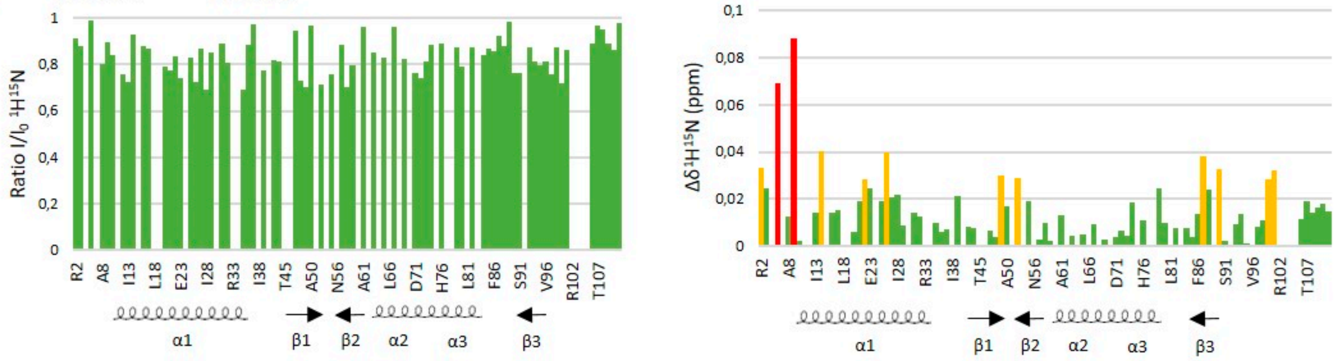

Supplementary Figure S6

Helicase Loading on foDNA

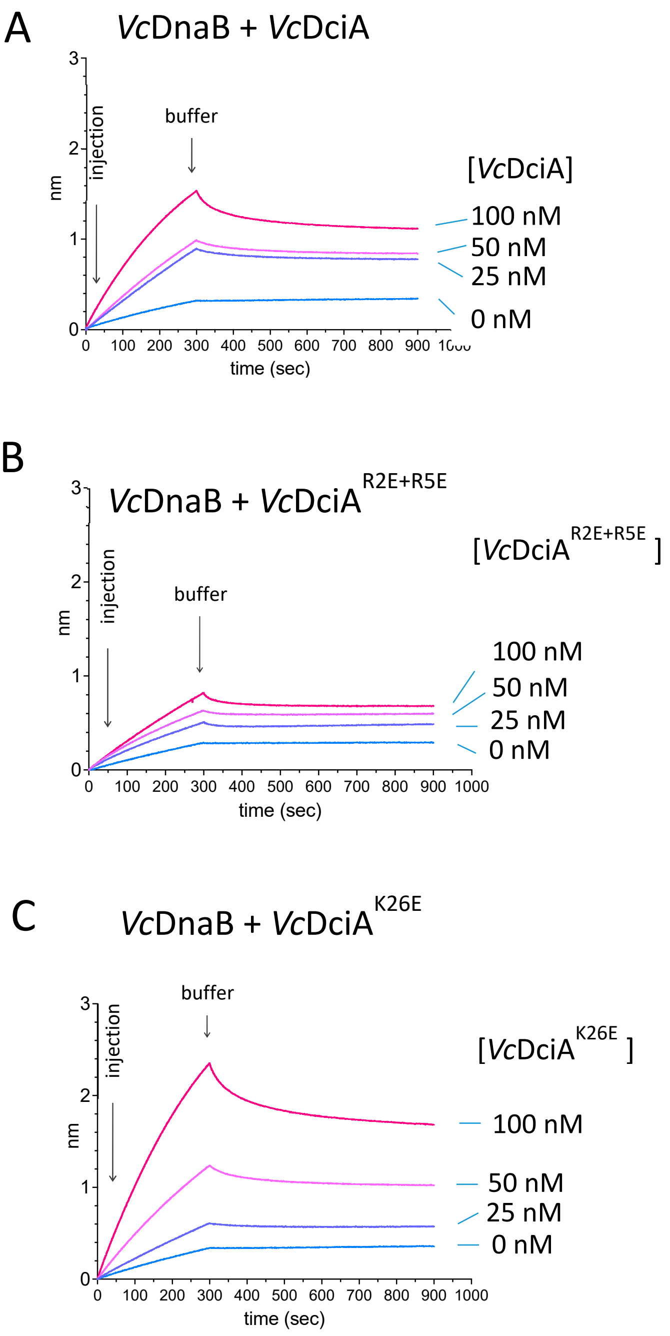

Supplementary Figure S7

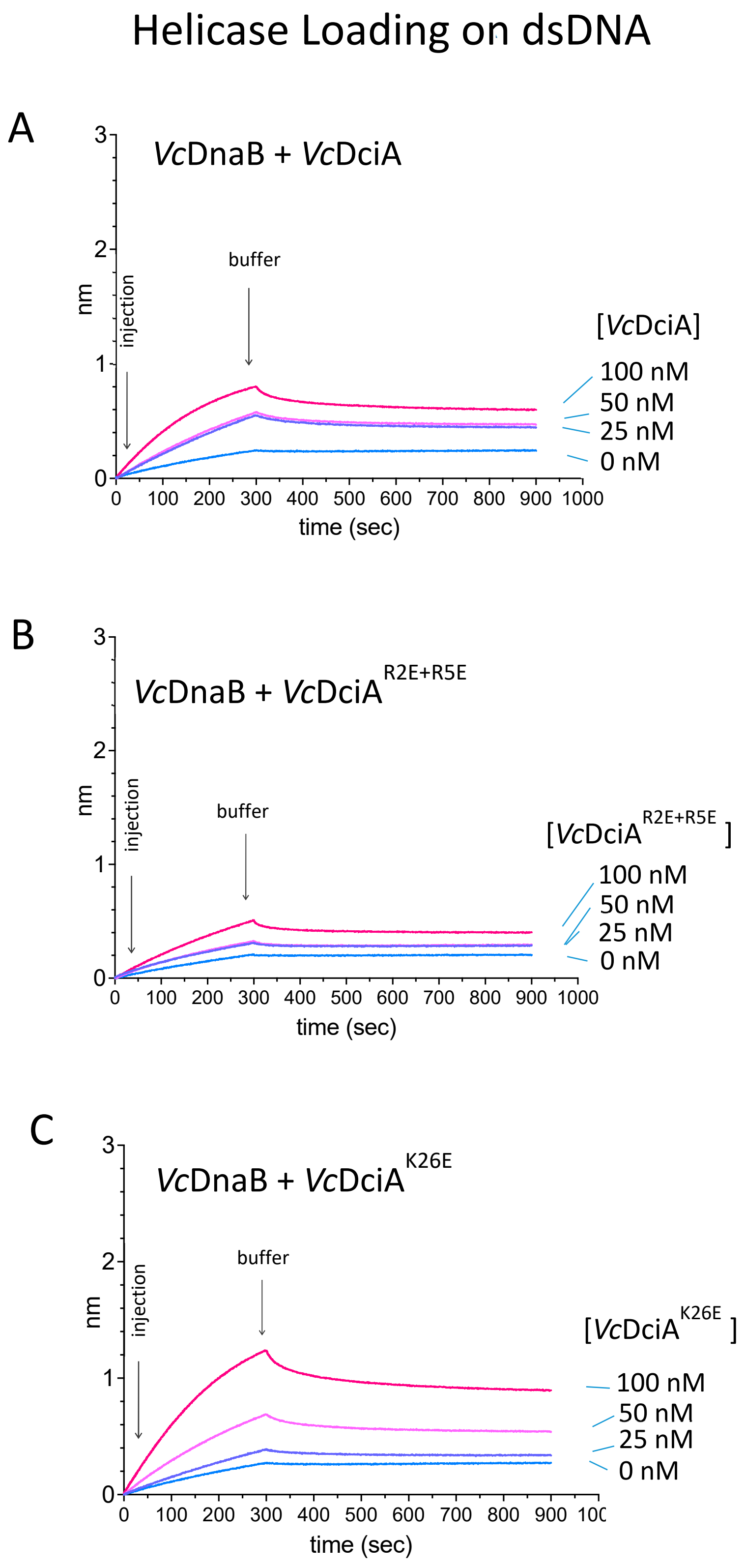

Supplement: Supplementary file 1 [file ijms-24-01427-s001.zip › ijms-2111522-supplementary.pdf]
